# The involvement of collagen family genes in tumor enlargement of gastric cancer

**DOI:** 10.1038/s41598-022-25061-0

**Published:** 2023-01-03

**Authors:** Hui Sun, Yufeng Wang, Shentao Wang, Yikui Xie, Kun Sun, Shuai Li, Weitong Cui, Kai Wang

**Affiliations:** 1 Department of Gastroenterology, Zibo First Hospital, Zibo, 255200 China; 2 Basic Medical Department, Qilu Medical University, Zibo, 255300 China; 3 Key Laboratory of Biomedical Engineering & Technology of Shandong High School, Qilu Medical University, Zibo, 255300 China

**Keywords:** Cancer, Computational biology and bioinformatics, Gastroenterology, Oncology

## Abstract

Extracellular matrix (ECM) not only serves as a support for tumor cell but also regulates cell–cell or cell–matrix cross-talks. Collagens are the most abundant proteins in ECM. Several studies have found that certain collagen genes were overexpressed in gastric cancer (GC) tissues and might serve as potential biomarkers and therapeutic targets in GC patients. However, the expression patterns of all collagen family genes in GC tissue and their functions are still not clear. With RNA sequencing (RNA-Seq) data, microarray data, and corresponding clinical data obtained from TCGA, GTEx, and GEO databases, bioinformatics analyses were performed to investigate the correlation between the expression patterns of collagen family genes and GC progression. We found that quite many of the collagen family genes were overexpressed in GC tissues. The increase in mRNA expression of most of these overexpressed collagen genes happened between T1 and T2 stage, which indicates the significance of collagens in tumor enlargement of GC. Notably, the mRNA expressions of these differentially expressed collagens genes were highly positively correlated. The elevated expression of a large number of collagen genes in early T stage might greatly change the composition and structure organization of ECM, contributing to ECM remodeling in GC progression.

## Introduction

GC is among the commonest malignant tumors related to the digestive system, ranking fifth for incidence and fourth for mortality globally, with over one million newly diagnosed cases and approximately 76,900 deaths reported in 2020^1^. Recent notable findings show an increase in the incidence of both cardia and non-cardia GC among young adults (aged < 50 years) in both low-risk and high-risk countries^2,3^. Late diagnosis, high degree of malignancy and early metastasis are three reasons for poor prognosis^4,5^. Although progress has been made in the prevention and treatment of this disease, GC remains an aggressive and poorly understood malignancy with a heterogeneous presentation and tumor biology. Thus, understanding the clinical and molecular characteristics of GC is of great importance for effective diagnosis and treatment.

Collagen is the most abundant and versatile extracellular matrix protein that promotes cell growth and forms the structural basis of tissues and organs^6^. Since the discovery of collagen II, 28 collagen types with different α chains encoded by 44 genes have been identified^7^. High expression of some collagen-encoding genes has been reported being associated with poor prognosis and progression of certain types of cancers^8–14^. Additionally, a couple of collagen genes, such as COL1A1, COL1A2, COL10A1, COL11A1 and COL12A1, were found overexpressed in GC tissues and may act as potential prognostic biomarkers and immune-associated therapeutic targets in gastric cancer^5,12,15–20^. However, most of these studies focused on expression and prognostic analysis of specific collagen genes. The correlation between the expression of collagen genes and GC progression remains to be clarified.

Previous studies on differential expression of collagen genes between tumor and normal tissues of GC mainly used methods of microarray^4,5,15,17,21,22^ or quantitative real time PCR^16,19,20^, lacking of analysis on RNA-Seq data. As a landmark cancer genomics program, TCGA sequenced and molecularly characterized a great many cases of various kinds of primary cancer samples. Due to the high variation in mRNA expression, differentially expressed genes (DEGs) identified using a larger sample size are more reliable in RNA-Seq analysis^23,24^. The number of GC samples in TCGA is much larger than that in any single study in other databases, and RNA-Seq is more accurate than the microarray technology; however, the expression patterns of collagen family genes in GC samples in TCGA has not been systematically explored.

In this study, we conducted a systematic analysis on the expression patterns of the whole collagen family and the correlation between the expression of collagen genes and GC progression. Bioinformatics analyses, including differential expression analysis, survival analysis, gene ontology (GO) and Kyoto Encyclopedia of Genes and Genomes (KEGG) enrichment analysis, spearman correlation analysis and protein–protein interaction (PPI) network analysis, were carried out to illuminate the involvement of collagen family genes in GC. Collectively, our works mainly explored the significance of collagen genes in tumor progression, especially tumor enlargement, of GC, and may help illuminate the potential mechanism of tumor progression and design novel strategies on targeted GC therapy.

## Methods

### Acquisition of RNA-Seq, microarray and clinical data

Raw RNA-Seq read count expression data and clinical data of all available GC tumor and adjacent normal tissues were obtained from TCGA. To ensure sample consistency, only primary solid tumor tissue samples and solid normal tissue samples were included. If a tissue sample was sequenced more than once, repeated data were excluded. After exclusion, the number of tumor and normal samples in GC datasets was 373 and 32, respectively. RNA-Seq data of 175 normal gastric tissue samples in GTEx database (https://www.gtexportal.org/) and GSE84437 dataset containing microarray data and corresponding clinical information of GC tumor tissue samples from 433 patients in GEO database (https://www.ncbi.nlm.nih.gov/geo/) were also obtained.

### Tissue specimens and immunohistochemistry (IHC)

Nine pairs of human gastric tumor and adjacent normal tissues were obtained from patients undergoing surgical resection at Zibo First Hospital, an affiliated hospital of Qilu Medical University (Zibo, China). The diagnosis of GC was confirmed by pathological examination. No patients had received anti-tumor therapy prior to surgery. All human specimens were obtained with the informed consent of patients. The study was approved by the ethics committee of Qilu Medical University and Zibo First Hospital and performed in accordance with the Declaration of Helsinki. Tissue samples were routinely fixed and embedded in paraffin. COL4A5 antibody (PA5-119042) was purchased from Thermo Fisher Scientific (Shanghai, China). SP Kit (Broad Spectrum) (SP0041) for immunohistochemical staining and detection was obtained from Solarbio (Beijing, China). Immunohistochemistry was conducted according to the instructions provided by the manufacturers. Images were acquired under a microscope (BX51, Olympus, Japan).

### Differential expression analysis

All analyses in this study were done in R (v 4.0.2) with different R packages. Differential expression analyses were done with the *edgeR*^25^ (v 3.30.3) and the *limma*^26^ packages. Trimmed-mean M values (TMM) normalization was performed to normalize the counts among different samples, and the exact test was applied for determining differential expression. Genes with false discovery rate (FDR) < 0.05 were considered as DEGs. Differential expression of the collagen family genes between GC and normal groups, as well as between the T1 stage and other three T stages, was also analyzed with Wilcoxon tests. The associations between expression of the collagen family genes and clinicopathologic parameters were analyzed with Wilcoxon and Kruskal tests. P < 0.05 was considered statistically significant. Boxplots and Heatmaps showing the distribution of the expression of the collagen family genes under different conditions were visualized using *ggpubr* (v 0.4.0) and *ComplexHeatmap*^27^ (v 2.4.3), respectively. For unified RNA-Seq data from GTEx and TCGA, differential expression analyses of all collagen genes between tumor and normal samples of GC were analyzed using the online web server of Gene Expression Profiling Interactive Analysis (GEPIA, http://gepia.cancer-pku.cn/).

### Survival analysis

Both the correlation between the mRNA expression level of collagen genes and overall survival (OS), and the correlation between clinical characteristics and OS, were tested by Kaplan–Meier method. When analyzing the correlation between collagen gene expression and OS, the hazard ratio (HR) of each collagen gene was defined as the hazard in the high expression group divided by the hazard in the low expression group and the cut-off value of mRNA expression of each collagen gene was set as its median value. In addition, HR > 1 and HR < 1 implied that higher expression of a particular collagen gene was associated with worse and better overall survival, respectively. HR was calculated by the univariate and multivariate Cox regression model using the *survival* (v 3.2-11) package. Survival curves were plotted using *survminer* (v 0.4.9) package. Log-rank tests were used to analyze the differences between survival curves, and P < 0.05 was considered statistically significant.

### Spearman correlation analysis

Correlations of mRNA expression between different collagen genes in GC samples were analyzed and plotted using *corrplot* (v 0.90) and *PerformanceAnalytics* (v 2.0.4) packages. Spearman correlation coefficient (PCC) values were calculated using normalized and Log2 transformed RNA-Seq read count data or microarray data.

### GO terms and KEGG pathway enrichment analysis and PPI network

DEGs between samples of the T1 stage and T2 stage were subjected to GO function analysis. KEGG pathways was explored by the gene sets enrichment analysis (GSEA) which was conducted by ranking all genes according to fold change and then calculating an enrichment score for each gene-set (pathway)^28–30^. Adjusted P < 0.05 was considered to be statistically significant for GO and KEGG pathway analysis. Both GO and GSEA were conducted using *clusterProfiler*^31^ (v 3.16.1) package. Genes involved in a particular enriched pathway were visualized by the *pathview* (v 1.28.1) package, and functional enrichment results obtained from GSEA were visualized by *enrichplot* (v 1.8.1) package.

The Search Tool for the Retrieval of Interacting Genes (STRING) database were used to construct the PPI network, and the cut-off criterion as confidence score was set at 0.40^32^. The results of protein–protein interactions were then subjected to Cytoscape (v 3.7.1) to visualize the network^33^. Next, in Cytoscape, the *cytohHubba*^34^ plugin was used to filter the top10 hub genes by the Maximal Clique Centrality (MCC) method, and the *Molecular Complex Detection* (*MCODE*)^35^ plugin was utilized to identify highly interconnected clusters in the PPI network with default settings.

## Results

### Differential expression of collagen genes in tumor and normal tissues

By far, the collagen superfamily is found to be comprised of 28 collagen types encoded by 44 genes (see Supplementary Table S1 online). Differential expression results analyzed between all GC and normal tissue samples showed that the mRNA expression levels of 34 collagen genes changed, of which 22 were up-regulated and 12 were down-regulated in tumor tissues (see Supplementary Fig. S1 online). Differential expression of collagen genes was also analyzed with Wilcoxon tests; however, the results were slightly different from those obtained using *edgeR*. The expression of six differentially expressed collagen genes identified using *edgeR*, namely COL4A4, COL6A2, COL8A2, COL9A3, COL11A2, and COL13A1, were not significantly different by Wilcoxon test. When unified RNA-Seq data of GC from GTEx and TCGA were used for differential expression analysis, the differential expression patterns of 36 out of 44 collagen genes were consistent with the results obtained when using the RNA-Seq data from TCGA alone (see Supplementary Fig. S2 online).

### Correlation between mRNA expression of collagen genes and clinical characteristics

**Figure 1:**
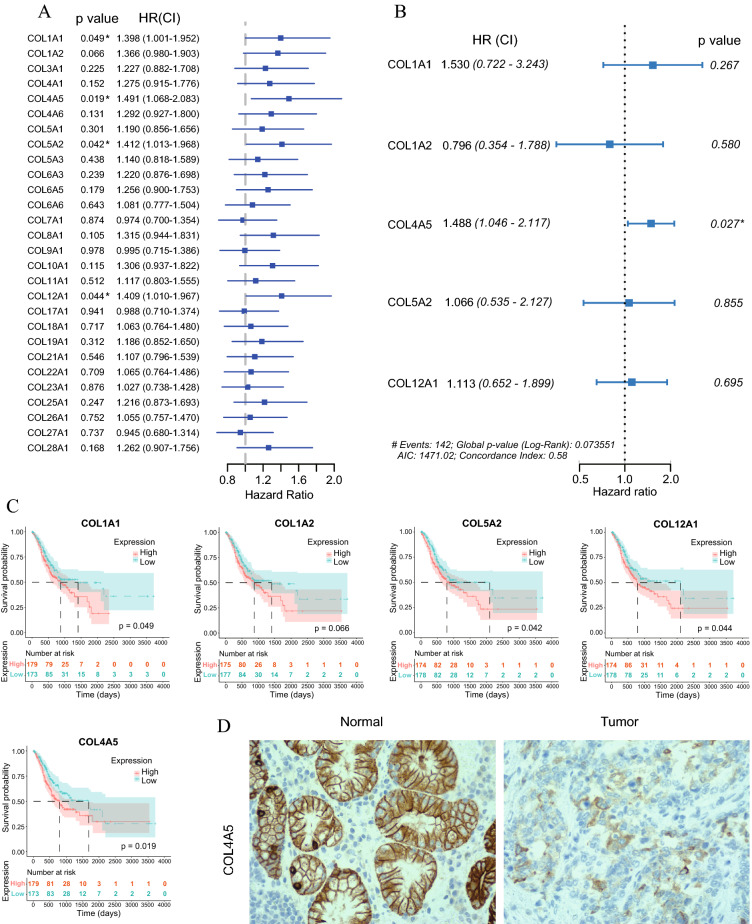
Correlations between the mRNA expression of different collagen genes and OS. (**A**) Univariate analysis. (**B**) Multivariate analysis for genes with p < 0.1 in the univariate analysis. (**C**) Kaplan–Meier survival curves for the five collagen genes with p < 0.1 in the univariate analysis. (**D**) Representative images of immunohistochemical staining for COL4A5 expression in GC tumor and normal tissues (400×). Unless specified, throughout this essay, the statistical significance was annotated by the number of stars (*: P-value < 0.05; **: P-value < 0.01; ***: P-value < 0.001; **** P < 0.0001).

To study the effect of high mRNA expression of collage genes on OS, correlations between high mRNA expression of the 28 differentially expressed collagen genes and OS were studied using univariate and multivariate analyses. As shown in Fig. 1A, although the HR of most of the 28 collagen genes was > 1.0 (Between 1.0 and 1.5), only the high expression of COL1A1, COL4A5, COL5A2, and COL12A1 was significantly related to OS. Collagen genes with P < 0.1 in the univariate analysis were further screened in multivariate analysis, and COL4A5 was found to be significantly related to OS (Fig. 1B). The survival curves of the 5 collagen genes with P < 0.1 in the univariate analysis were demonstrated in Fig. 1C. As shown in Fig. 1D, COL4A5 was detected in both GC tumor and normal tissues, but the expression was significantly higher in GC normal tissues (P = 0.02).

**Figure 2:**
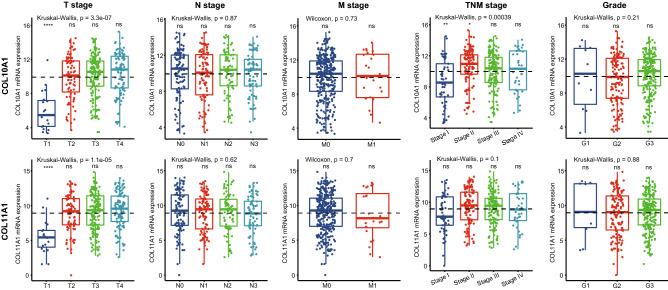
Differential expression of COL10A1 and COL11A1 between different stages of GC. The mean mRNA expression value of each gene in all tumor samples was set as reference for all the comparisons.

The differential expression results in Supplementary Fig. S1 demonstrated that COL10A1 and COL11A1 had the largest fold changes (130 and 64, respectively). Many previous studies had found that these two genes were overexpressed in tumor tissues^4,5,15–22^ and were correlated with T stage^5,15^. We analyzed the mRNA expression of COL10A1 and COL11A1 between different T, N, M, and TNM stages, as well as between different grades. As shown in Fig. 2, the mRNA expression of these two genes was up-regulated significantly only in the early stage of T stage, and no significant difference was seen between different N and M stages.

In the TNM system, the T refers to the size and extent of the main tumor, the N refers to the number of nearby lymph nodes that have cancer, and the M refers to whether the cancer has metastasized. The results indicate that the high expression of these two genes is closely related to tumor enlargement, but not to the lymph node metastasis and distant metastasis. By analyzing the mRNA expression of all collagen genes in different T stages (see Supplementary Fig. S3 online), another 23 collagen genes, which included most of the differentially expressed collagen genes between the GC and normal tissues, were found to share the same expression patterns as COL10A1 and COL11A1.

Univariate analyses of different tumor stages and grades for OS demonstrated that poor overall survival was significantly correlated with higher T, N, M, and TNM stages (Table 1). Notably, the HR of T stages was much larger than those of N, M, and TNM stages. Although higher tumor grades showed greater HR, the correlation between GC grade and OS was not significant.

**Table 1:** Univariate analyses of different tumor stages and grades for OS in GC patients.

Variables	n (%)	HR (95% CI)	P
**T stage**			
T1	18 (4.83%)	1 (Reference)	
T2	74 (19.84%)	6.475 (0.878–47.746)	0.067
T3	161 (43.16%)	9.467 (1.314–68.195)	0.026
T4	95 (25.47%)	9.772 (1.343–71.121)	0.024
Not available	25 (6.70%)		
**N stage**			
N0	104 (27.88%)	1 (Reference)	
N1	95 (25.47%)	1.560 (0.969–2.512)	0.067
N2	71 (19.03%)	1.472 (0.871–2.487)	0.149
N3	71 (19.03%)	2.430 (1.506–3.923)	0.0003
Not available	32 (8.58%)		
**M stage**			
M0	314 (84.18%)	1 (Reference)	
M1	22 (5.9%)	2.389 (1.346–4.242)	0.003
Not available	37 (9.92%)		
**TNM Stage**			
Stage I	48 (12.87%)	1 (Reference)	
Stage II	110 (29.49%)	1.629 (0.825–3.215)	0.160
Stage III	145 (38.87%)	2.294 (1.207–4.358)	0.011
Stage IV	34 (9.12%)	4.080 (1.977–8.421)	0.0001
Not available	36 (9.65%)		
**Grade**			
G1	9 (2.41%)	1 (Reference)	
G2	126 (33.78%)	1.648 (0.400–6.790)	0.489
G3	208 (55.76%)	2.140 (0.527–8.699)	0.287
Not available	30 (8.04%)		

### Differential expression of collagen genes between T1 and other T stages

**Figure 3:**
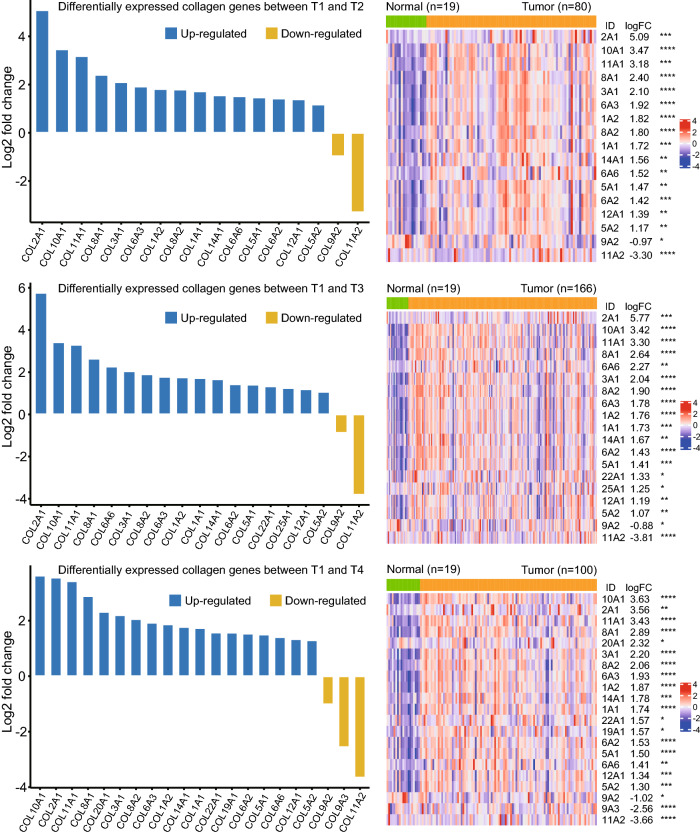
Differentially expressed collagen genes between T1 and other three T stages. Samples of the T1 stage were served as control group for all the differential expression analyses.

According to the results above, the transition from T1 to T2 might be a critical step for ECM evolution. To analyze the collagen genes involved in tumor progression and the genes that interacted with collagen genes, differential expression analyses were performed between T1 stage and other three T stages (T2, T3, and T4, respectively). As shown in Fig. 3, the differentially expressed collagen genes and their expression patterns for the three differential expression analyses were similar. Expression of differentially expressed collagen genes was also checked with Wilcoxon tests.

The differential expression results of some genes, such as COL2A1 and COL11A2, were different between the two methods. E*dgeR* analysis demonstrated that COL2A1 was significantly up-regulated in samples of T2, T3, and T4 stages with quite large fold change values, while the results of Wilcoxon tests showed that the differential expression of COL2A1 between T1 and T3, and also between T1 and T4, was not significant. Similarly, e*dgeR* analysis revealed that COL11A2 was significantly down-regulated in T2, T3, and T4 stages, but the difference was not significant in Wilcoxon tests. By examining the read count values of COL2A1 and COL11A2, the large fold change values obtained using *edgeR* were caused by a few extreme outlier expression values, which was consistent with previous studies^23,24^.

Among the 15 collagen types that are up-regulated in the T2 stage, COL1A1, COL1A2, COL2A1, COL3A1, COL5A1, COL5A2, and COL11A1 are fibril-forming collagens, COL12A1 and COL14A1 are fibril-associated collagens, while COL8A1, COL8A2, COL10A1 are network-forming collagens^36^. Normally, the expression of these genes is tissue-specific and mutations in these genes are associated with many diseases^36^. Although the up-regulated expression of these genes has been observed in GC and many other cancers, the functions of these genes in tumor progression remain largely unclear. COL9A2, down-regulated in the T2 stage, constitutes the α chain of type IX collagen which is a fibril-associated collagen. Type IX collagen is an important component of cartilage, which makes up much of the skeleton during early development. However, the function of the COL9A2 in tumor progression has remained elusive.

Solid tumors are often associated with excessive tissue fibrosis due to increased synthesis, crosslinking and deposition of fibrillar collagen, mainly collagen type I and III^37^. Fibrous ECM proteins are major components of the tumor microenvironment and play a major role in tumor tissue stiffness^38,39^. Increase in matrix stiffness and alignment has been shown as a hallmark in many cancers, such as breast cancer, pancreatic cancer, and prostate cancer^40,41^. Tumor cells sense the increased ECM stiffness through specific mechanoreceptors and become more aggressive^42^. Therefore, the up-regulation of a great number of fibril-forming collagens, network-forming collagens, and fibril-associated collagens at T2 stage might alter ECM homeostasis and promote tumor growth.

### Correlation analysis of differentially expressed collagen genes in tumor tissue

Although several collagen genes have been reported to be differentially expressed between different T stages, to the best of our knowledge, the correlation between the expression of collagen genes have not been reported before. Differential expression analysis by *edgeR* and Wilcoxon test identified 14 collagen genes that were differentially expressed between T1 and all the other three T stages. Compared with T1 stage, the mRNA expressions of the 14 collagen genes in T2, T3, and T4 stages were all up-regulated. Correlations between the mRNA expression of the 14 collagen genes in GC samples was analyzed by spearman correlation analysis.

**Figure 4:**
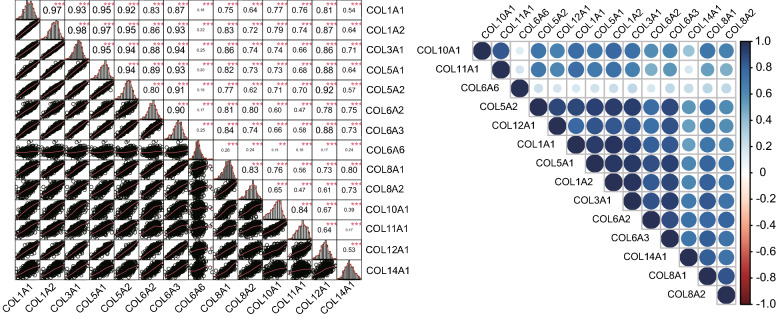
Correlation among the 14 up-regulated collagen genes between T1 and T2 stage in GC tumor tissue. Within the figure, the left part represented the scatter plot and matching correlation values from − 1 to 1, whereas the right part showed the correlation strength in different color and the statistical significance in different size.

As exhibited in Fig. 4, significant and positive relationships existed among all of the 14 genes. High correlations were found among COL1A1, COL1A2, COL3A1, COL5A1, COL5A2, COL6A2, and COL6A3, with PCC values larger than or close to 0.9. Interestingly, the correlations between COL6A6 and other genes were weak (PCC < 0.3). The correlation analysis of these 14 collagen genes based on GSE84437 dataset in GEO database gave similar results (see Supplementary Fig. S4 online). Correlation among the 9 down-regulated collagen genes in GC tumor was also analyzed, and the results showed that a strong positive correlation existed between the mRNA expression of COL4A5 and COL4A6 (see Supplementary Fig. S5 online).

### GO and KEGG pathway enrichment analysis and PPI network analysis

A total of 2551 DEGs (|log_2_FC|> = 1, FDR < 0.05) including 1968 significantly up-regulated genes and 583 significantly down-regulated genes were identified between T1 and T2 stages, and these genes were classified according to GO term. The top-ranked terms for BP, CC, and MF were extracellular matrix organization, collagen-containing extracellular matrix, and extracellular matrix structural constituent, respectively, and many of the significantly enriched terms were related to ECM, indicating active changes of ECM in early stages of GC (see Supplementary Fig. S6 online). The 7 KEGG pathways enriched by GSEA that involved collagen genes and the enrichment scores (ES) for the 7 pathways can also be found as Supplementary Fig. S6 online.

**Figure 5:**
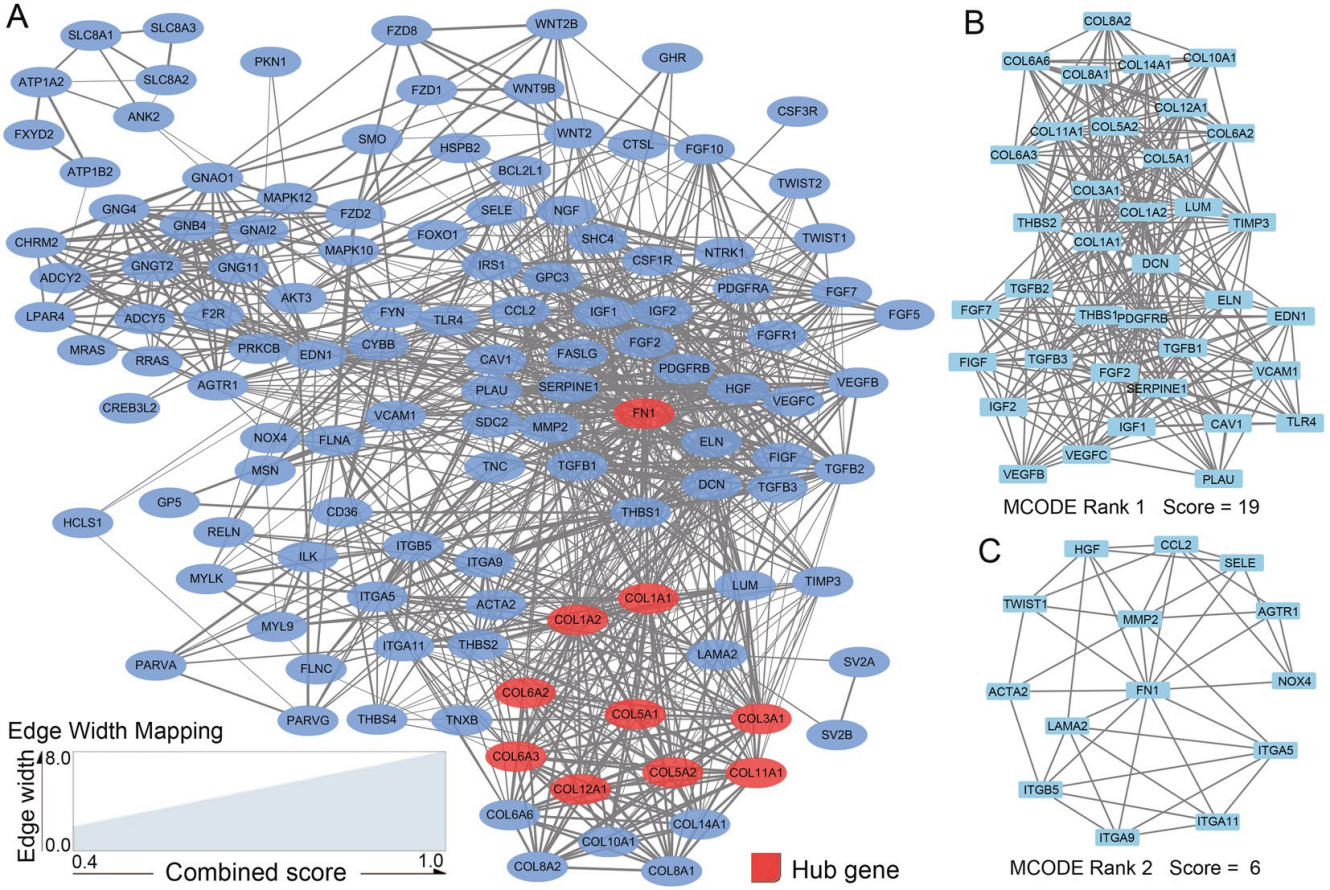
PPI network analysis. (**A**) The PPI network of DEGs enriched in the 7 KEGG pathways involved with collagen genes and the top 10 hub genes identified by cytohubba using the MCC method in Cytoscape. Three disconnected nodes were hidden in the network. (**B**, **C**) The top 2 clusters identified by MCODE in Cytoscape.

Together 281 genes were enriched in the 7 pathways. As GSEA took into account all the genes included in differential expression analysis, rather than only the DEGs, differential expression of the enriched genes was further checked with differential expression results and Wilcoxon test. The resulting 125 significantly differentially expressed genes (All up-regulated in GC tissues of T2 stage) were used for the following interaction network analysis. As shown in Fig. 5A, the PPI network including 125 nodes and 1120 edges was constructed by Cytoscape software, based on STRING database. The top 10 genes with the largest number of adjacent nodes, namely COL1A1, COL1A2, COL3A1, COL5A1, COL5A2, COL6A2, COL6A3, COL11A1, COL12A1, and Fibronectin 1 (FN1), were selected as hub genes. Figure 5B demonstrated the sub-network between 14 differentially expressed collagen genes and 23 other DEGs, while Fig. 5C exhibited the sub-network between FN1 and 13 other DEGs.

## Discussion

Previous studies^5,17,19–22^ mainly focused on expression changes of certain collagen genes between tumor and normal tissues, but differential expression of collagen genes during tumor progression was seldom studied. We found that the mRNA expression of many collagen genes underwent a sharp increase between T1 and T2 stage. Nevertheless, the overexpression of most collagen genes was not significantly correlated with OS, indicating that the high expression of most collagen genes might be a necessary step in tumor progression of GC, irrelevant to OS. The high expression of COL4A5 was significantly related to poor OS, which implied that COL4A5 could act as an independent prognostic marker for GC, consistent with the findings of Zeng et al. focusing on the COL4A family^43^.

Xiao et al.^44^ reported that COL4A5 was indispensable in cancer development by using COL4A5-deficient mouse model, and epithelial COL4A5 supported cancer cell proliferation, while endothelial COL4A5 was essential for efficient tumor angiogenesis. Based on the findings of Xiao et al., it is reasonable that high expression of COL4A5 indicates a worse prognosis of GC. But it is still puzzling why the expression of COL4A5 is down-regulated in GC tumor tissues.

As components of basement membrane, type IV collagens are essential in the maintenance of tissue integrity and proper function^44^. COL4A5 is a component of α3α4α5 trimers, while COL4A5 and COL4A6 form α5α5α6 trimers^36^. Both α3α4α5 and α5α5α6 are minor type IV collagens. Since the expression of COL4A5 is decreased in GC tumor tissue and a strong positive correlation exists between expression of COL4A5 and COL4A6, the formation and quantity of minor type IV collagens in GC tumor might be affected. Therefore, further research is needed to explore collagens perhaps with spatial or architectural analysis.

Both tumor stage and grade can be used to determine a patient's prognosis. By analyzing the correlation between clinical characteristics and OS, we found that different staging methods (T, N, M, and TNM) of the TNM staging system were all significantly correlated with OS, but the HR values of T stage were much large than those of other staging methods, indicating that early detection and early treatment was important for good prognosis. Generally, a higher tumor grade implies a worse prognosis, but the clinical data of GC in TCGA showed no significant correlation between advanced grade and OS.

Based on the correlation between clinical characteristics (T stage, N stage, M stage, TNM stage, and grade) and OS, and also the expression features of collagen genes between different tumor stages and different tumor grades, the T staging method which refers to the size and extent of the primary tumor might better correspond to the ECM changes at molecular level in GC. The up-regulation of the mRNA expression of a large number of collagen genes between T1 and T2 stage implies that high expression of collagen genes is closely linked with tumor enlargement of GC.

The pathological difference between T1 and T2 is a tumor with or without invasion to muscularis propria (MP). Mucosa is the innermost layer of the stomach where nearly all stomach cancers start^45^, while MP is a thick layer of smooth muscle beneath submucosa and mucosa. It has been reported that tumor cells, tumor-associated fibroblasts, and tumor-associated macrophages work in concert to modulate the tumor microenvironment and exhibit excessive deposition of collagen and collagen-transforming enzymes, particularly at the invasive front^46,47^. Tumor cells were also found to act as an organizer of stromal collagen^48^. Thus, as GC tumor progresses, the distinct difference in composition and structure between MP, submucosa, and mucosa might induce tumor microenvironment changes, including changes in quantity, type, and alignment pattern of the collagen family, to facilitate MP invasion.

Not only were many collagen genes overexpressed in tumor progression between T1 and T2 stages, but also the mRNA expression of these collagen genes, such as COL1A1, COL1A2, COL3A1, COL5A1, COL5A2, COL6A2, and COL6A3, was highly positively correlated. Since the mRNA expression of these genes was highly correlated, targeted expression suppression of one gene might destroy the synergistic effect of multiple genes. Therefore, these highly correlated genes might act as potential therapeutic targets in gastric cancer.

ECM is a dynamic structure that is constantly remodeled to control tissue homeostasis^49^ and the relationship between ECM remodeling and cancer progression has also been reported^50–53^. Our findings revealed the increased mRNA expression of 15 collagen genes in GC tumor tissue in the early stage of GC. Additionally, elevated protein expression of certain collagen genes has also been reported in GC tumor tissue^5,15,21,22^. Various collagen types form polymeric assemblies and have complex interactions with other proteins^53^. The increased production and density of ECM confers the tumor microenvironment with tumor-promoting and cell-spreading properties^38^. Fibrillar collagen accumulation in the stroma creates a very dense network of ECM fibrillar proteins, which gradually leads to tissue stiffening^53^. The high stiffness restricts the motility of immune cells, preventing their antitumor activity^54,55^, and imposes new mechanical constraints to resident and tumor cells, forming a tumorigenic niche that leads to altered mechanotransduction pathways and supports tumor progression and invasion^42^. As the most abundant proteins in ECM, the increased expression of a large number of collagen genes might greatly change the composition and structure organization of ECM, contributing to ECM remodeling in the progression of GC, which was also supported by the results of GO and KEGG analyses.

PPI analysis revealed that the differentially expressed collagen genes had complex interactions with other DEGs between T1 and T2. The 10 hub genes in the PPI network contained 9 collagen genes and FN1 which was a ubiquitous ECM glycoprotein that played vital roles during tissue repair^56^. The top 2 sub-networks identified by MCODE demonstrated that collagen genes and FN1, as different types of ECM components, were closely interacted with different sets of genes. What was intriguing was that both MMP2, which was a collagenase degrading extracellular matrix proteins, and TIMP3, which was a metalloproteinase inhibitor that could irreversibly inactivate MMP2, were up-regulated between T1 and T2, along with the up-regulation of several collagen genes.

Collagens play structural roles and contribute to mechanical properties, organization, and shape of tissues^36^. The composition and structure of ECM in tumors might change along with the expression changes of collagens. The evolution of the thick collagen network surrounding cancer cells increases the risk of developing metastases, making it a potential indicator of cancer stages^38^. It has been reported that tumor-associated collagen signatures (TACSs) are distinct in tumors of different stages and TACS-3, which describes radially aligned collagen fibers oriented perpendicular to the tumor, is seen in advanced stage tumors^38,57–59^. The overexpression of certain collagens might contribute to GC development by promoting tumor cell proliferation, migration, and invasion^12,20^. Hsp47, a collagen-specific molecular chaperone encoded by the *SERPINH1* gene, is indispensable for molecular maturation of collagen. The elevated mRNA expression of *SERPINH1* in tumor tissues of GC (see Supplementary Fig. S2 online) indicates trouble in collagen maturation^60^.

Taken together, the high expression of many collagen genes in early T stage of GC may cause drastic changes of ECM and contribute to GC progression. Although what we found indicates the significance of the collagen genes in tumor enlargement of GC, many issues, such as the types of cells that overexpress collagen genes in GC tissue considering that tumors are a heterogenous mixture of different cell populations^61^, whether the overexpressed collagen genes form complex or work alone, and the roles of overexpressed collagens, still need to be further clarified.

## Supplementary Information

Supplementary Information.

## Data Availability

The datasets supporting the conclusions of this study are available in TCGA database (https://www.cancer.gov/tcga), GTEx database (https://www.gtexportal.org/), and GEO database (https://www.ncbi.nlm.nih.gov/geo/).
