# Supplementary material for: The involvement of collagen family genes in tumor enlargement of gastric cancer
Source: Sci Rep. 2023 Jan 3;13:100. doi: 10.1038/s41598-022-25061-0 (PMC9810739; doi:10.1038/s41598-022-25061-0)
Supplement: Supplementary file 1 — Supplementary Information. [file 41598_2022_25061_MOESM1_ESM.pdf]

The involvement of collagen family genes in tumor enlargement of gastric cancer

Hui Sun[1,†], Yufeng Wang[1,†], Shentao Wang[2], Yikui Xie[1], Kun Sun[1], Shuai Li[2], Weitong Cui[3,*], Kai Wang[2,*]

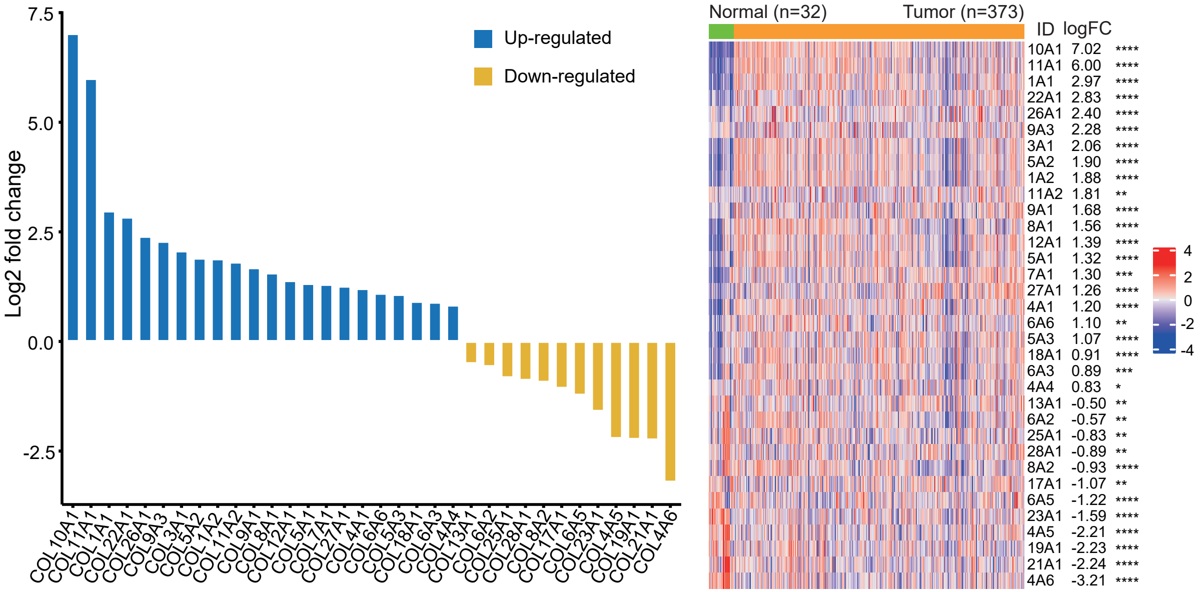

Supplementary Figure S1. Differential expression analysis of collagen family genes between GC and normal tissues. Normal tissue samples were chosen as control group. * P < 0.05, ** P < 0.01, *** P < 0.001, and **** P < 0.0001.

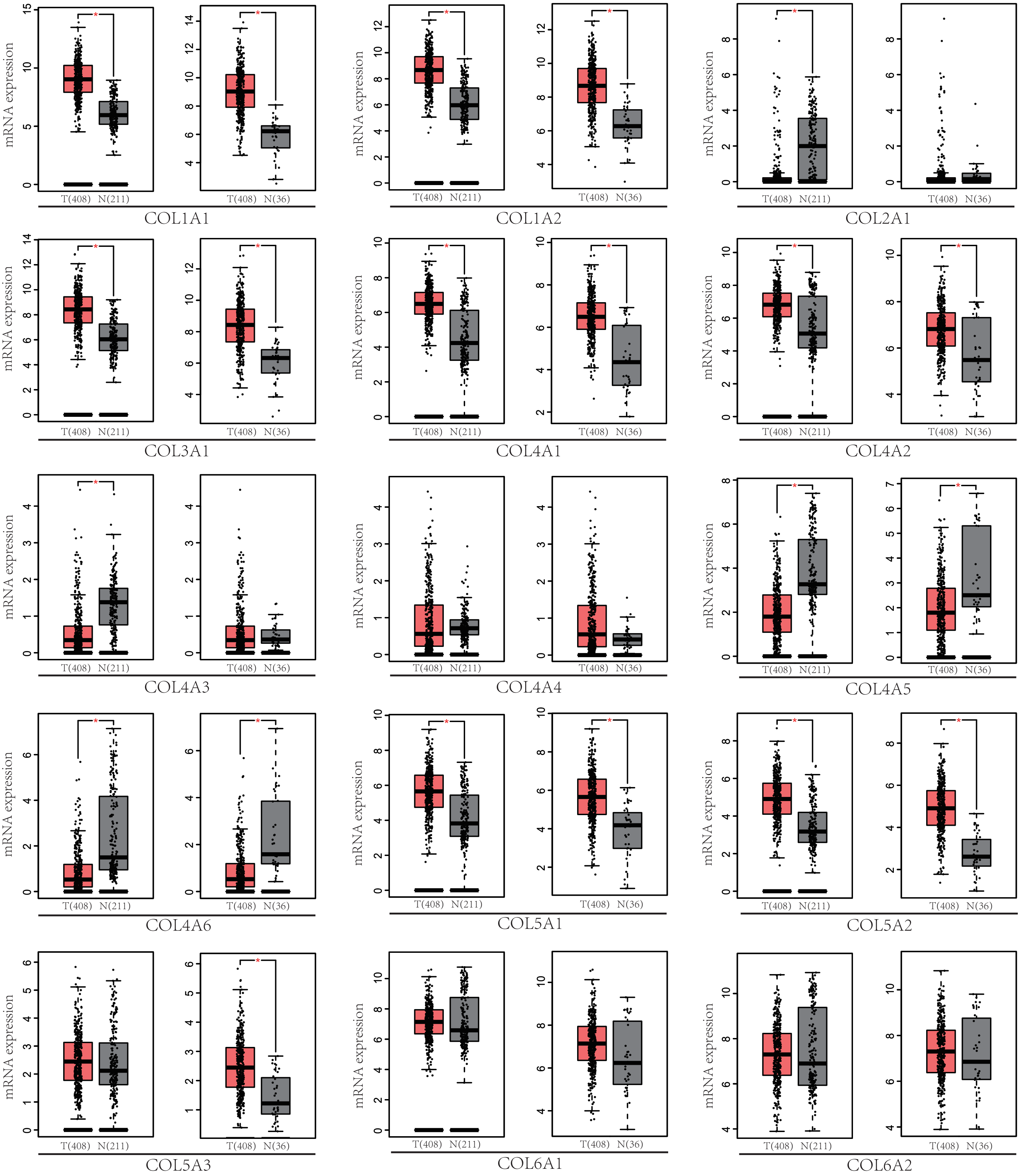

Supplementary Figure S2. Differential expression analysis of all collagen genes and *SERPINH1* in GC at transcriptional level (GEPIA). For each gene, differential expression analysis between tumor and normal samples was performed twice with the same tumor tissue samples from TCGA. If the normal samples were from TCGA alone, the differential expression results were exhibited in the right boxplot. If the normal samples were from GTEx and TCGA together, the results were shown in the left boxplot. N, normal gastric tissues. T, tumor tissues. The numbers enclosed in parentheses after “T” or “N” referred to the number of tissue samples in each group. *, P<0.01.

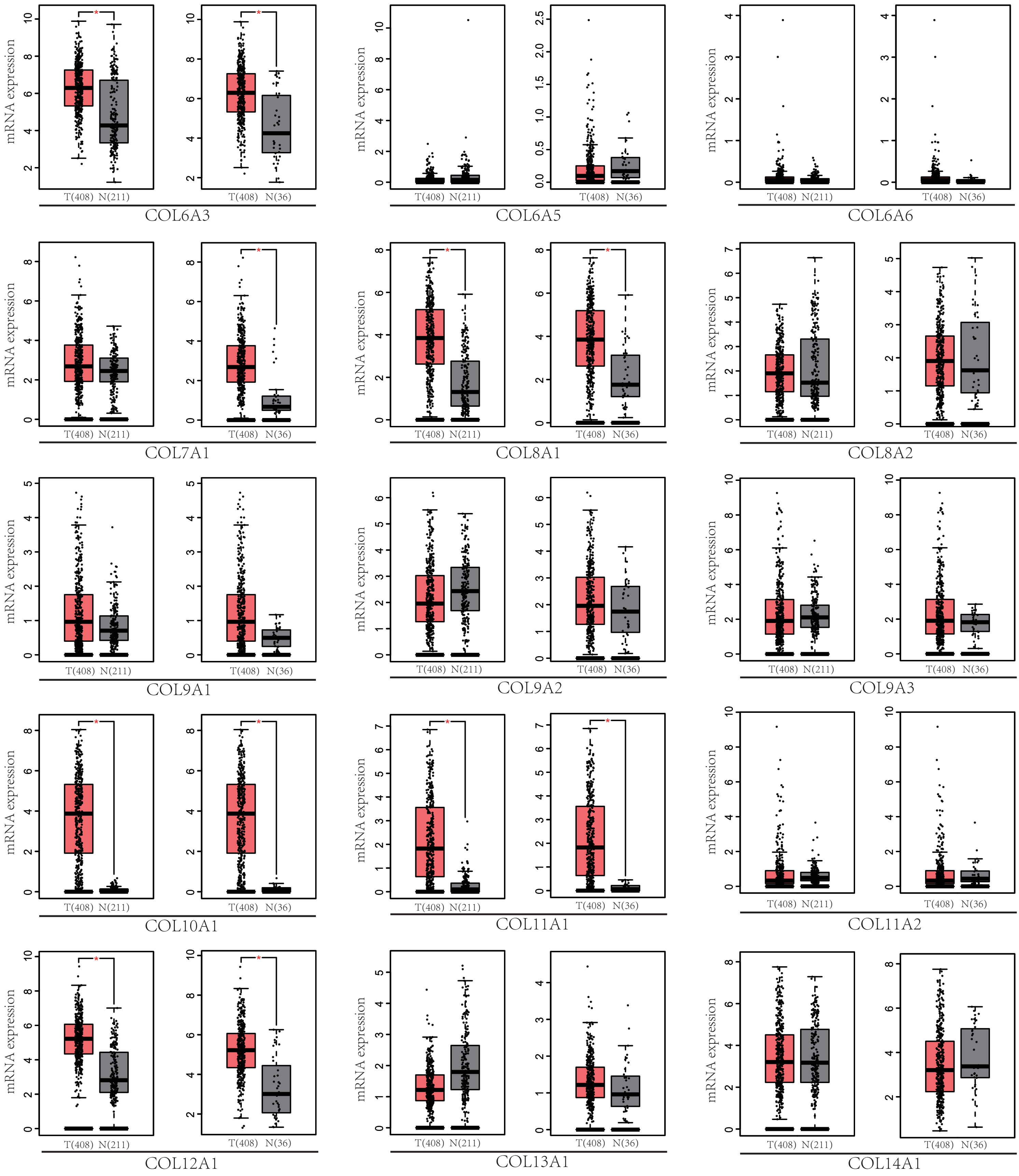

Supplementary Figure S2. (Continued.)

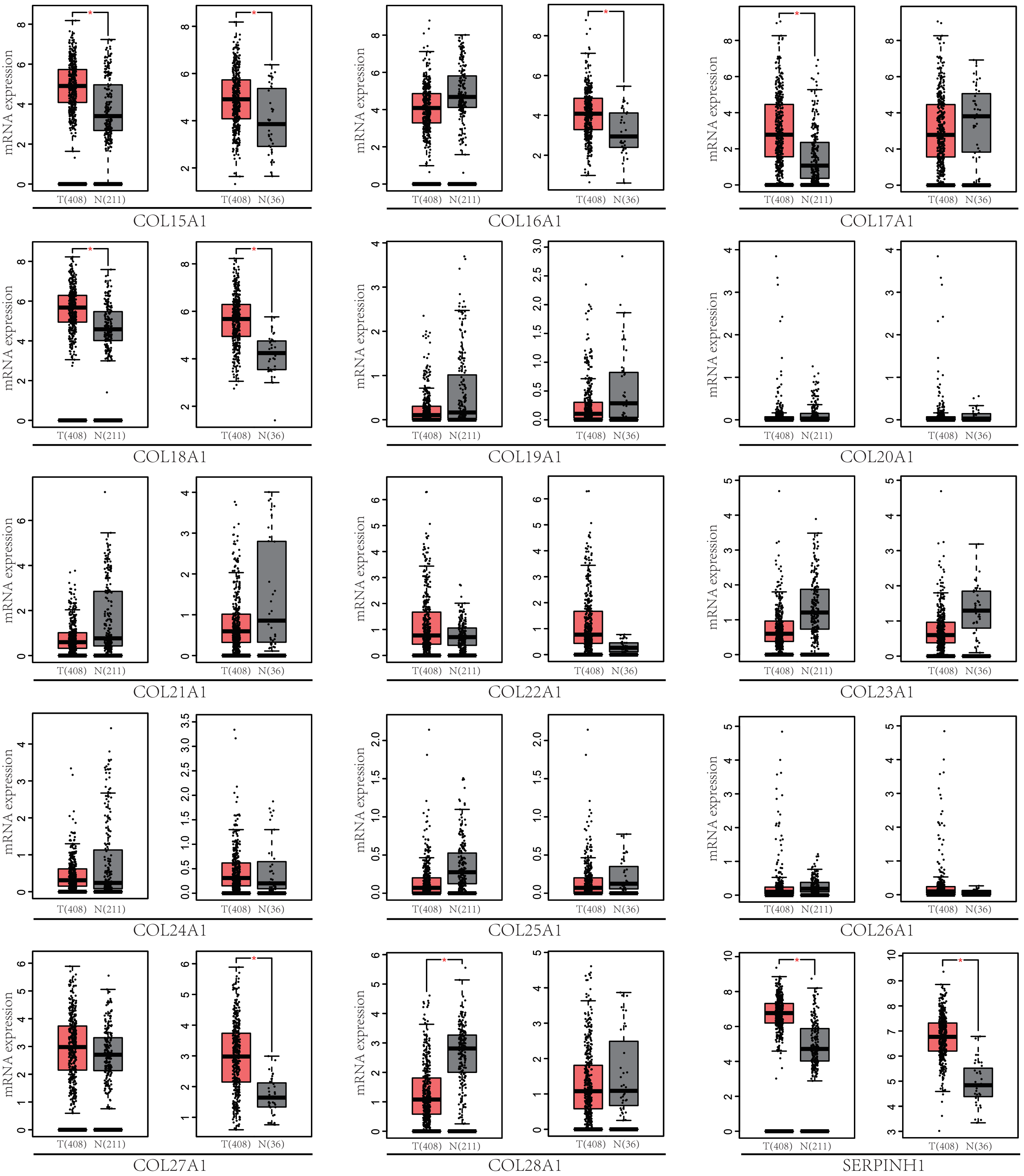

Supplementary Figure S2. (Continued.)

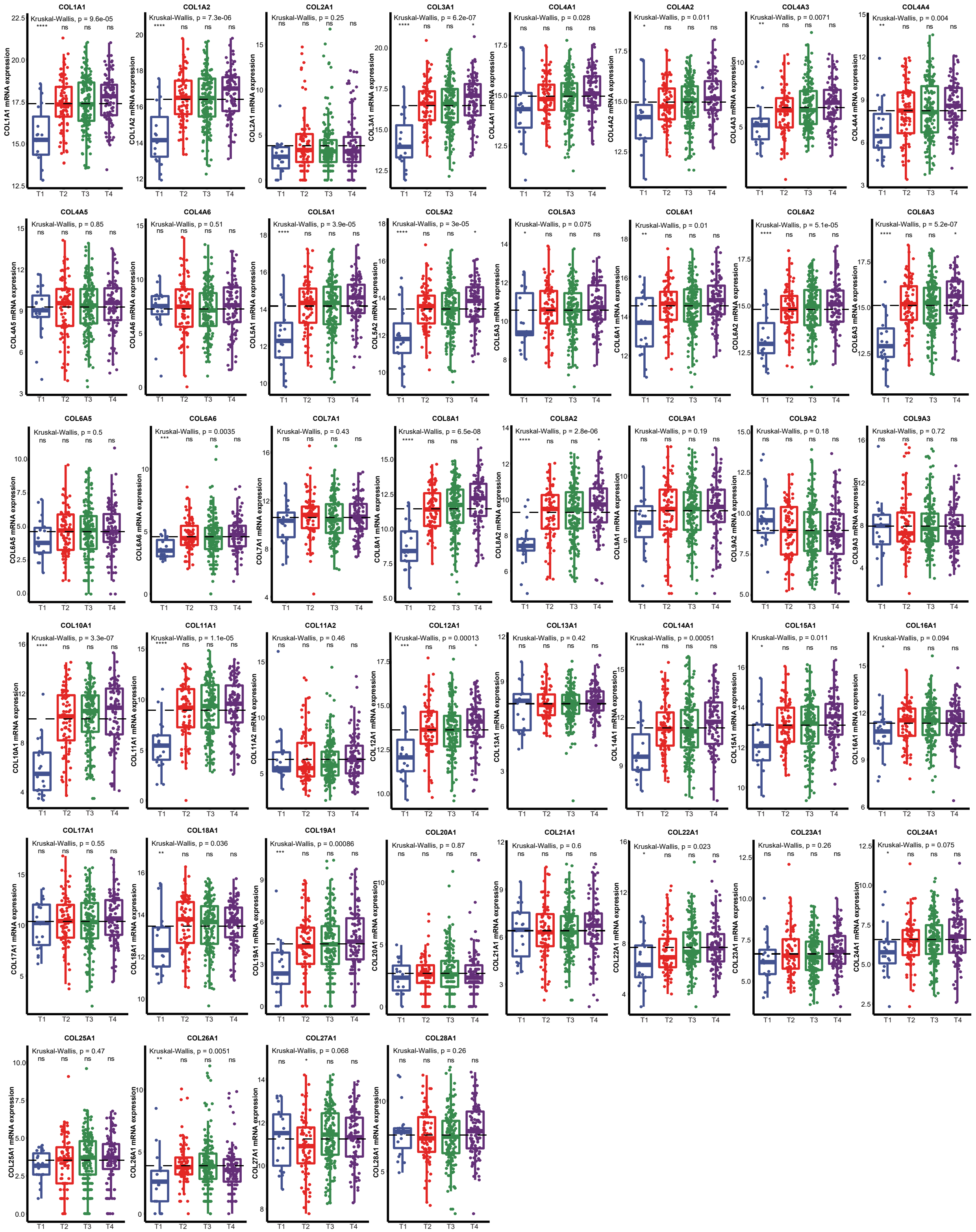

Supplementary Figure S3. Differential expression of collagen family genes between different T stages of GC.

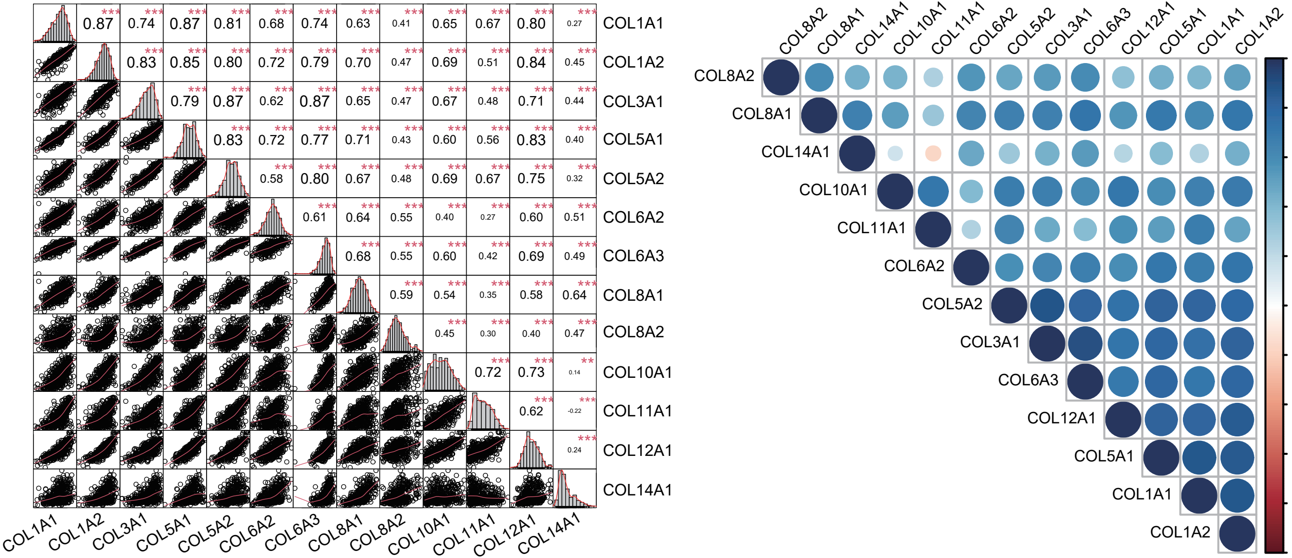

Supplementary Figure S4. Correlation among the 14 up-regulated collagen genes based on tumor samples of GSE84437 dataset. Within the figure, the left part represented the scatter plot and matching correlation values from -1 to 1, whereas the right part showed the correlation strength in different color and the statistical significance in different size.

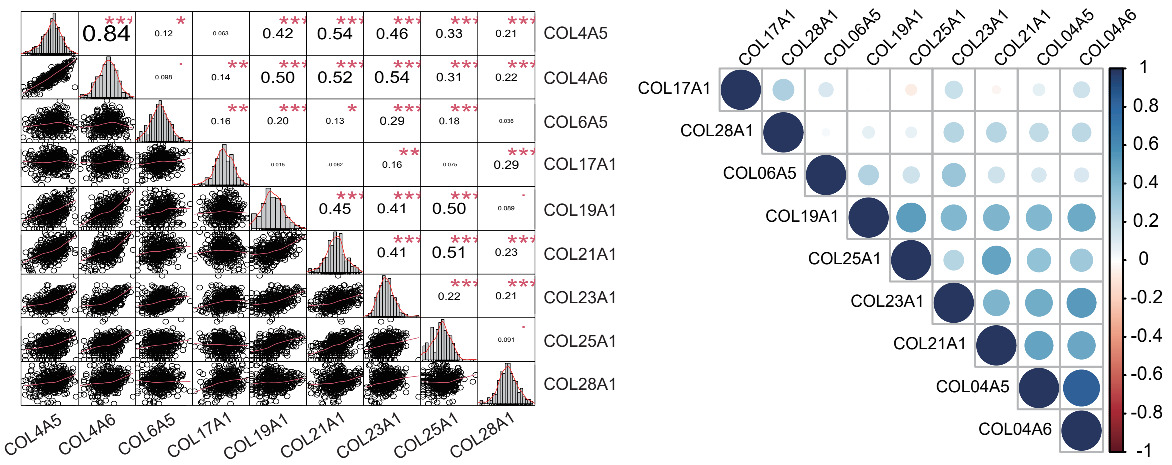

Supplementary Figure S5. Correlation among the 9 down-regulated collagen genes in GC tumor samples of TCGA. Within the figure, the left part represented the scatter plot and matching correlation values from -1 to 1, whereas the right part showed the correlation strength based on color and the statistical significance in different size.

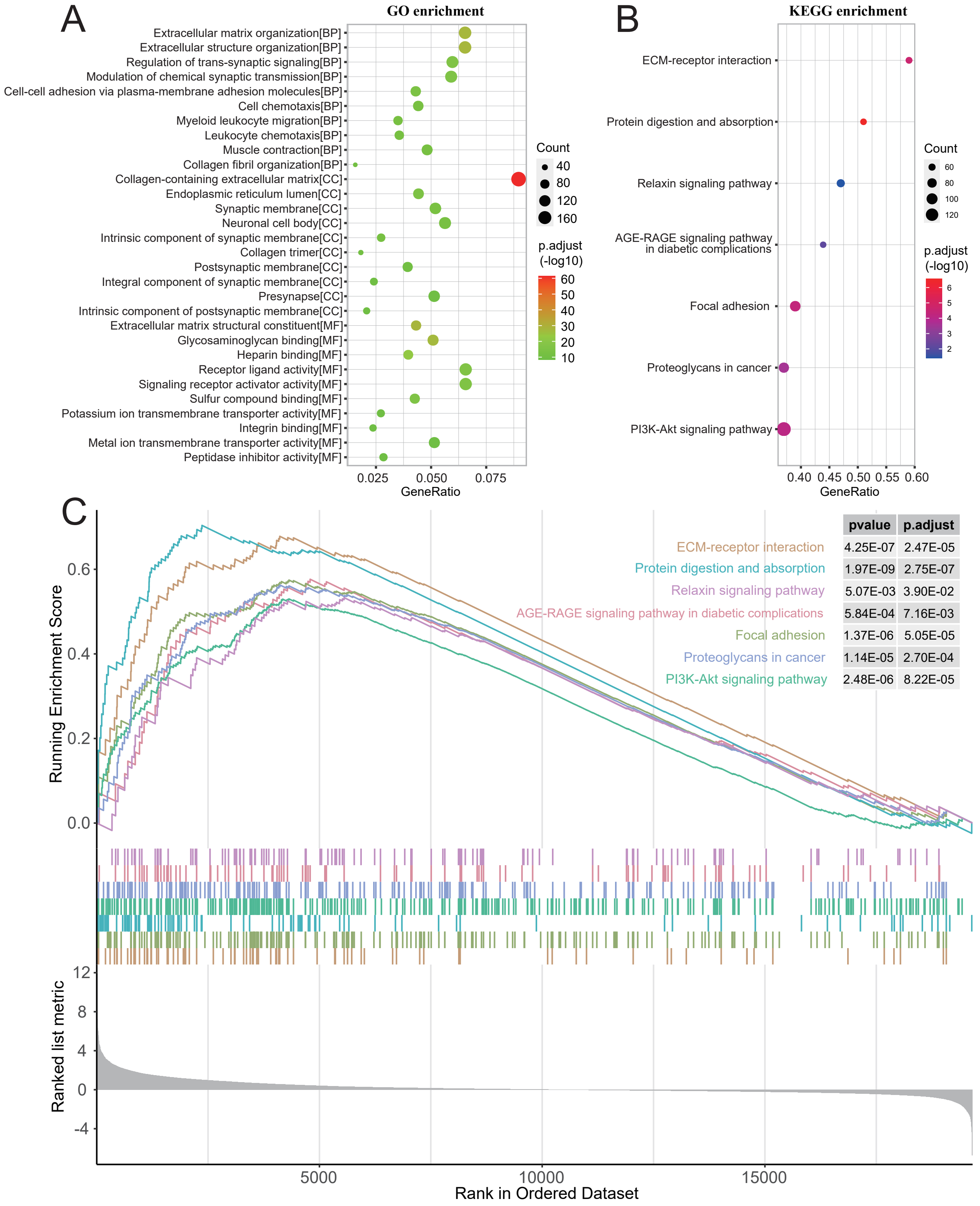

Supplementary Figure S6. Functional enrichment analysis of DEGs between T1 and T2 stages. (A) GO analysis. (B) The 7 KEGG pathways involving collagen genes. (C) Enrichment plot for the 7 KEGG pathways involving collagen genes in GSEA.

Supplementary Table S1. ID of the 44 genes that encode 28 types of collagen.

ENSEMBL	ENTREZID	SYMBOL
ENSG00000108821	1277	COL1A1
ENSG00000164692	1278	COL1A2
ENSG00000139219	1280	COL2A1
ENSG00000168542	1281	COL3A1
ENSG00000187498	1282	COL4A1
ENSG00000134871	1284	COL4A2
ENSG00000169031	1285	COL4A3
ENSG00000081052	1286	COL4A4
ENSG00000188153	1287	COL4A5
ENSG00000197565	1288	COL4A6
ENSG00000130635	1289	COL5A1
ENSG00000204262	1290	COL5A2
ENSG00000080573	50509	COL5A3
ENSG00000142156	1291	COL6A1
ENSG00000142173	1292	COL6A2
ENSG00000163359	1293	COL6A3
ENSG00000172752	256076	COL6A5
ENSG00000206384	131873	COL6A6
ENSG00000114270	1294	COL7A1
ENSG00000144810	1295	COL8A1
ENSG00000171812	1296	COL8A2
ENSG00000112280	1297	COL9A1
ENSG00000049089	1298	COL9A2
ENSG00000092758	1299	COL9A3
ENSG00000123500	1300	COL10A1
ENSG00000060718	1301	COL11A1
ENSG00000204248	1302	COL11A2
ENSG00000111799	1303	COL12A1
ENSG00000197467	1305	COL13A1
ENSG00000187955	7373	COL14A1
ENSG00000204291	1306	COL15A1
ENSG00000084636	1307	COL16A1
ENSG00000065618	1308	COL17A1
ENSG00000182871	80781	COL18A1
ENSG00000082293	1310	COL19A1
ENSG00000101203	57642	COL20A1
ENSG00000124749	81578	COL21A1
ENSG00000169436	169044	COL22A1
ENSG00000050767	91522	COL23A1
ENSG00000171502	255631	COL24A1
ENSG00000188517	84570	COL25A1
ENSG00000160963	136227	COL26A1
ENSG00000196739	85301	COL27A1
ENSG00000215018	340267	COL28A1
